# Low Levels of Detectable Urine and Stool GIPs in Children with Celiac Disease on a Gluten-Free Diet

**DOI:** 10.1097/PG9.0000000000000323

**Published:** 2023-06-09

**Authors:** Maxwell Horton, Katherine L. Olshan, Elizabeth Gleeson, Stephanie Regis, Taylor Morson, Zackary J. Hintze, Maureen M. Leonard, Jocelyn A. Silvester

**Affiliations:** From the * Celiac Research Program, Harvard Medical School, Boston, Massachusetts, USA; † Division of Gastroenterology, Hepatology and Nutrition, Boston Children’s Hospital, Harvard Medical School, Boston, Massachusetts, USA; ‡ Division of Pediatric Gastroenterology and Nutrition, MassGeneral Hospital for Children, Harvard Medical School, Boston, Massachusetts, USA; § Mucosal Immunology and Biology Research Center, MassGeneral Hospital for Children, Boston, Massachusetts, USA; ∥ Department of Pediatrics, Harvard Medical School, Harvard University, Boston, Massachusetts, USA.

## Abstract

**Objectives:**

This study examines the prevalence of detectable gluten immunogenic peptides (GIPs) as a proxy for gluten exposure in children with celiac disease on a gluten-free diet in the United States, as estimated by gluten breakdown products excreted in urine and stool.

**Methods:**

Urine and stool samples were collected in 3 settings (home, gastroenterology clinic, and endoscopy) for pediatric participants (ages 6–21 years old) across 2 medical centers. Commercial ELISA assays were used to quantify the GIPs in each sample.

**Results:**

GIPs were detected in 4 out of 44 (9.1%) of stool samples and 6 out of 125 (4.8%) of urine samples provided by 84 children. These samples were collected across all settings, and most participants (70%) were asymptomatic at the time of sample collection. For the urine samples collected at the time of endoscopy, all subjects found to have persistent enteropathy had no detectable GIPs (0/12).

**Discussion:**

GIPs provide an additional method for screening for gluten exposures in individuals with celiac disease on a gluten-free diet, and may be used across multiple settings. We found a low detection rate of GIPs in children. Our finding of undetectable GIPs in individuals with persistent enteropathy may be expected of a single determination under close observation or represent a lack of gluten exposure within the detection window. More research is needed to understand the dynamics of gluten absorption and excretion in the US pediatric population.

**What Is Known**

- Assessing adherence to a GFD is an inexact science.
- Clinical symptoms, patient self-report, expert dietitian assessment, celiac serology, and duodenal biopsy are not reliable measures of GFD adherence.
- Tests that measure gluten immunogenic peptides in urine and stool have recently revealed that gluten exposure may be more pervasive than is generally believed.

**What Is New**

- We found GIP in 9.1% of stool samples and 4.8% of urine samples, across multiple testing environments, in children with celiac disease on a GFD for at least 6 months.
- In comparison to previous pediatric studies in Europe, which reported a 13%–25% stool detection rate, our study at 2 Celiac Disease Centers in the United States had a lower detection rate.
- In the subset of patients with persistent enteropathy, none had detectable levels of GIPs at the time of endoscopy.

## INTRODUCTION

Celiac disease (CeD) is a chronic autoimmune condition characterized by responsiveness to gluten ingestion in genetically susceptible individuals. Currently, the only treatment for CeD is a strict, lifelong gluten-free diet (GFD) (1). Yet, this treatment is very difficult to comply with because gluten is a component of many staple foods and is not always listed as an ingredient on food labels (2,3). Overall, 36%–55% of patients who report strict adherence to a GFD have persistent histologic damage (3–5). In the pediatric population, 1 in 5 patients has persistent intestinal enteropathy, possibly due to ongoing gluten exposure (6).

The current method for evaluating GFD adherence involves a combination of dietary assessment either by a Registered Dietitian or with questionnaires (eg, Biagi questionnaire), serological tests, and clinical symptoms, which is highly subjective and notoriously inaccurate (3–5,7). In recent years, immunoassays have been developed to directly measure gluten immunogenic peptides (GIPs), such as the immunodominant 33-mer, which are resistant to gastric and pancreatic proteases and ultimately cause mucosal inflammation (5,7). The immunoassays use monoclonal antibodies to detect GIP excretion in urine and stool samples. Thus, these tests could provide an objective and noninvasive assessment of gluten exposure. The aim of our study is to determine the prevalence of detectable GIPs in the urine and stool as a proxy for gluten exposure in children with CeD on a GFD in the United States.

## METHODS

### Recruitment

Two cohorts of children, 1 from Massachusetts General Hospital (MGH) and 1 from Boston Children’s Hospital (BCH), were studied after receiving the proper ethical approval. All subjects included in the study had elevated serum tissue transglutaminase antibody levels (tTG) Immunoglobulin A (IgA) and were diagnosed with CeD according to the histologic assessment of duodenal biopsies using Marsh-Oberhuber criteria (8).

Children at MGH were recruited at the time of initial diagnostic endoscopy or at a clinically indicated follow-up endoscopy as part of an ongoing pediatric biorepository, which recruits children ages 2–21 years old. Written informed consent was obtained. The inclusion of patients aged 21 years or younger in the cohort is in accordance with the United States of America Federal Food, Drug, and Cosmetic Act, which defines pediatric patients as those 21 years old or younger at the time of diagnosis. For the purposes of the cohort utilized for this article, only samples from follow-up endoscopy and from children aged 6–21 years were used. All participants followed standardized institutional guidelines in preparation for the endoscopy (no milk or solid foods since 11 pm the night before the endoscopy, and no clear liquids for the 2 hours before their arrival time). Participants were blinded as to what urine samples would be used for. At the time of endoscopy, participants and/or their parents completed a validated GFD compliance questionnaire (9) which is based on a 1-week dietary recall where participants were classified as either adherent or nonadherent. Additionally, participants and/or their parents completed the validated gastrointestinal symptom rating scale which assesses gastrointestinal symptoms over the last week (10,11). Subjects undergoing endoscopy during this study were classified as either having CeD in remission (Marsh 0–2) or as having persistent enteropathy (Marsh 3).

At BCH, children 6–18 years old with biopsy-confirmed CeD on a GFD for at least 6 months were recruited either by phone or at the time of outpatient gastroenterology follow-up appointments. Upon giving signed informed consent, participants provided urine and stool samples at the time of these visits, and they were notified about what these samples would be tested for. Those who enrolled in a clinical trial (NCT03462979) were randomized (1:1) to either blinded sample collection or self-testing to provide real-time feedback. Home sample collection occurred at random intervals prescribed by the study team and participants were notified on their scheduled day of sample collection so as to limit the impact of anticipated observation on behavior. Each participant (or their parent) ranked their level of adherence to a GFD on a categorical scale based on the Gluten-Free Eating Assessment Tools with either no gluten or rare accidental gluten less than once per month considered adherent (12). At the time of sample collection, parents completed the ObsRO Celiac Disease Daily Symptom Diary. The clinical trial was terminated early due to the COVID-19 pandemic.

### Tissue Transglutaminase Antibody Levels

IgA tTG at the time of diagnosis were processed at various outpatient laboratories. Values are reported here as multiples of the upper limit of normal (ULN) for the assay used.

For serum samples collected at the time of endoscopy, the tTG IgA level was measured at MGH using QUANTA Lite Rh-tTG IgA ELISA using INOVA Quanta Flash on BIO-flash (INOVA Diagnostics, San Diego, CA, USA) with a ULN of 20 U/mL. Samples collected at clinical visits were tested using EliA Celikey IgA (Thermofisher Scientific, Waltham, MA) with a ULN of 6.9 U/mL.

### Detection of GIP in Urine and Stool

Urine and stool samples collected during medical visits (either endoscopy or outpatient visits) were immediately frozen at −20ºC (MGH samples) or −80ºC (BCH samples) for storage. Home samples were stored in the participant’s home freezer and then transferred frozen to BCH for storage at −80ºC. The urine and stool samples were thawed completely before GIP testing using either the iVYCHECK GIP Stool or iVYCHECK GIP Urine (iVYDAL In Vitro Diagnostics, Biomedal SL) following manufacturer instructions. The iVYCHECK Reader immunochromatographic test cassette reader (iVYDAL Diagnostics, Seville, Spain) was used to quantify the amount of GIP for each sample. For the endoscopy cohort, each urine sample was run in duplicate.

Urine and stool samples collected at home had the GIP testing performed by the participant or their parent/guardian with a single-use Gluten Detect lateral flow assay (Biomedal SL, Seville, Spain) according to the manufacturer’s instructions. Participants reported a qualitative positive or negative response and sent a photograph to the study team. A positive result was indicated by the appearance of a red line in the test region along with a green control band.

## RESULTS

A total of 84 participants (64% female, median 13 years old) with at least 1 urine sample were included in the study (Table 1). Additionally, 22 of those participants (16 female, median 11 years old) had at least 1 stool sample collected. The Number of Participants row of Table 1 represents the number of participants that had samples collected in that setting (in endoscopy, in clinic, or at home) and sample type (urine or stool). Both the tTG IgA numerical value and normal cutoff value at diagnosis were available for 73 participants (median 10× ULN, range 1.4–28× ULN). The other 10 patients had elevated tTG IgA but multiples of the ULN could not be calculated because the result was recorded simply as “positive”, because the result exceeded the upper limit of the dynamic range of the assay used, or because testing had been performed at an outside laboratory and the limits of the dynamic range were not available. In these cases, the raw tTG IgA value exceeded the upper limit of all assays used in North America. Upon enrollment in the study, there was a median time of 24 months (range 6–168) on a GFD with 24 participants having an elevated tTG IgA with a median of 2.1× ULN (Table 1).

**TABLE 1.** Participant and sample characteristics

Location of sample collection	In endoscopy^*^	In clinic^†^		At home^†^		Total
	Urine	Urine	Stool	Urine	Stool	
Number of participants	42	42	18	13	4	84
Median age at enrollment (years)	16.2 (12.0–18.1)	12.0 (10.3–14.5)	10.6 (8.6–13.2)	13.3 (10.6–15.9)	11.9 (10.5–13.3)	13.2 (10.5–17.0)
Sex (females, % [n])	64 (27)	64 (27)	72 (13)	69 (9)	75 (3)	64.3
Diagnosed via endoscopy (yes, %)	95	100	100	100	100	98
tTG IgA at diagnosis (MULN) (median) (n = 73)	20 (18–25)	10.0 (4–19)	10.3 (6.9–19.0)	8.5 (6.9–19.0)	6.9 (6.2–10.2)	7.8 (6.9–25)
tTG IgA at enrollment (MULN) (median)	0.4 (0–1)	2.9 (2.0–5.7)	2.7 (1.5–4.1)	2.9 (2.4–7.1)	2.4 (2.2–14.3)	1.5 (0.3–3.5)
Median time on GFD (months)	24 (15–51)	35.8 (16.1–59.5)	37.2 (20.8–63.9)	21.8 (12.4–52.1)	26.3 (14.4–41.7)	24 (15–55.0)
Dietetic counselling (yes, %)	86	100	100	100	100	93
Adherence to GFD (yes, %)^*^	91^‡^	72^§^	71^§^	73^§^	50^§^	82
Number of samples	42	42	18	41	26	169
Samples with detectable GIPs (% (n))	2.4 (1)	7.1 (3)	5.6 (1)	4.9 (2)	11.5 (3)	5.9 (10)
Median GIP in samples with detectable GIPs (ng/mL)	12	^∥^	25	^∥^	30	25
Symptoms at sample collection (%)	50^¶^	34.5^#^	35.7^#^	50^#^	50^#^	43^#^

GFD = gluten-free diet; GIP = gluten immunogenic peptides; MULN = multiples of the upper limit of normal.

^*^Participants recruited at MGH.

^†^Participants recruited at BCH.

^‡^Adherence determined using GFDCQ (9).

^§^Adherence determined using Gluten-Free Eating Assessment Tools. Those who reported no gluten or rare accidental gluten exposure less than once per month were considered adherent.

^∥^Only qualitative testing was performed on these urine samples.

^¶^Symptoms determined using GSRS.

^#^ObsRO Celiac Disease Symptom Diary.

Overall, 4 out of 44 (9.1%) of stool samples and 6 out of 125 (4.8%) of urine samples had detectable GIPs (Table 1). The 10 samples with detectable GIPs were distributed among 8 patients, as 1 patient had 3 samples with detectable GIPs. Of the 4 stool samples with detectable GIPs, 3 were collected at home and 1 was collected in the clinic. One-third of the participants with detectable GIPs reported a rare accidental gluten exposure less than once a month and the other two-thirds reported no gluten exposure. Of the 6 urine samples with detectable GIPs, 3 were collected in the clinic, 2 at home, and 1 at the time of endoscopy. Most participants with detectable GIPs in the urine or the stool (cumulatively, 4/4 in clinic, 2/5 at home, and 1/1 in endoscopy) were asymptomatic before having these samples collected. Those who provided serial samples at home had a higher positivity rate per individual than those who provided samples at endoscopy or in the clinic only (30% vs 7%).

For the samples collected in the clinic or at home, 30 participants reported their adherence to the GFD, and all reported rare accidental exposure or no exposure. Of the 3 participants with a urine sample with detectable GIPs at the first visit, only 2 reported their level of gluten exposure and both said they had no gluten exposure. The participant with a stool sample with detectable GIPs at visit one self-reported no gluten exposure.

Of the 42 individuals selected after undergoing a follow-up endoscopy, 30 were in remission (Marsh 0–2), and 12 had persistent enteropathy (Marsh 3). Those in remission had been on a GFD for a median of 24 months (range 11–168) whereas those with persistent enteropathy had been on a GFD for a median of 17 months (range 11–168). For these individuals, 1 out of 30 in remission had urine with detectable GIPs (12 ng/mL), compared with 0 out of 12 with persistent enteropathy.

There was no statistically significant difference between participants with and without detectable GIPs in terms of age at enrollment, Body Mass Index, self-reported adherence to the GFD, time on GFD, tTG IgA at diagnosis, or tTG IgA at enrollment.

## DISCUSSION

In this multicenter study in the United States, we identified a lower rate of detectable gluten exposure using GIPs in both stool (9.1%) and urine (4.8%) in children with CeD on a GFD for at least 6 months when compared with previously published pediatric and adult studies of stool GIP (13%–25%) and urine GIP (5.4%–38%) (13–15). While the presence of gluten exposure for individuals with CeD following a GFD has been demonstrated in prior studies, our results are unique in that samples were collected in multiple settings, including endoscopy, clinic, and at home (13,14). Our findings support previous work showing that symptoms are not a reliable screen for gluten exposure as most participants with detectable GIPs did not report symptoms at the time of sample collection (12,16–18).

Multiple factors may explain our findings of a lower frequency of detectable GIPs in CeD patients on a GFD when compared with prior studies. First, the clinical context that the urine and stool samples were collected might have affected these results as those in the clinical trial knew that it was a study of dietary adherence that involved urine testing. Other studies investigating the kinetics of urinary excretion time of GIPs relative to gluten ingestion suggest a key window for GIP detection may be 3–9 hours postingestion (range 1–24 hours) (12,19,20). In our study, children who underwent endoscopy had avoided milk and solid foods since 11 pm the night before the endoscopy and clear liquids for 2 hours prior (as is standard practice at our institution for an endoscopy with general anesthesia), thus it is possible that GIP may have been excreted by some participants before urine collection in the endoscopy unit. This is in contrast with samples collected in the clinic where the participants may have eaten a meal or snack within a few hours before their visit. It is also possible that our results are indicative of improved GFD education and/or adherence among subjects. All samples for this study were collected through specialized celiac centers at MGH and BCH, where 93% of participants received dietetic counseling on the GFD. Finally, the majority of participants provided a single sample.

The degree to which repeat sampling may have affected our results is dependent upon the pattern of gluten exposure on a GFD, which is not well characterized. In our cohort of 17 persons who collected serial samples at home, 5 had a sample with detectable GIP, but only 1 had multiple samples with detectable GIP. Thus, we did not have “compliant” and “noncompliant” participants, rather more intensive sampling was more likely to detect gluten. This concurs with other studies where GIPs were detected more frequently when participants collected multiple samples (4,20,21).

Nevertheless, this study was designed to evaluate the practicality of screening for gluten exposures using GIPs in a clinical setting where a single collection is preferred despite potential detection limitations including the timing of GIP excretion postingestion. It is worth noting that while urine samples are more easily collected in a clinical setting, prior research has shown that the detection window for GIP in the stool (<24–>72 hours) is longer than the detection window in urine (3–36 hours) (3,12,20,22). Studies suggest that similar amounts of gluten exposure (>40 mg/day) can be detected with both urine and stool GIPs in samples collected within the appropriate postingestion timeframe (3,18,22). Our data suggest that more research is needed to understand the range of gluten excretion in children and the sensitivity and specificity of GIP testing before GIPs can be used as point-of-care tests in clinical settings.

It is noteworthy that none of the 12 patients undergoing follow-up endoscopy on a GFD with persistent enteropathy had detectable GIPs (in urine), especially given all participants were unaware that urine samples would be used to screen for gluten exposure. This is in contrast to prior studies, in which GIPs were detected in samples from 94% to 100% of participants who had duodenal mucosal damage on endoscopy (defined as Marsh 2 or Marsh 3) (3,4,23). A detectable GIP value with histologic evidence of active CeD might suggest that repeated gluten exposure was triggering the ongoing inflammation. It is possible that the children in our study were ingesting sufficient gluten to trigger intestinal inflammation, but that was below the limit of detection for urine GIPs.

The amount of gluten needed to elicit mucosal damage has not been well-defined but is known to be variable. Prior research suggests that 3 months of 50 mg per day of gluten ingestion is sufficient to cause mucosal inflammation in adults with CeD, which is much lower than the previously estimated mean (150–400 mg) and median (100–150 mg) daily gluten exposures extrapolated from GIP excreted in stool and urine test (24,25). It is also possible that testing multiple urine samples for GIPs would increase the sensitivity and utility of assessing dietary adherence in individuals with evidence of persistent enteropathy on endoscopy.

Alternatively, it is possible that individuals undergoing repeat endoscopy might have altered their dietary habits and behaviors in the short term (the window of detection of urinary gluten is 6–16 hours, cleared from the circulation and urine by 24 hours), thus avoiding gluten in the day(s) leading up to the procedure and explaining the negative single determination, though it is worth noting that these participants were unaware that their urine would be evaluated for gluten exposures (19). It is possible that they might have had more long-term gluten exposure that triggered the persistent enteropathy noted on pathology. This is consistent with the known lag between initiating a GFD and mucosal recovery (26). Additional research is needed to further understand both the utility of measuring GIPs to detect ongoing gluten exposure in children with persistent enteropathy and the kinetics of mucosal damage and recovery from intermittent gluten exposures on a GFD.

Strengths of our study include the multicenter collaboration, as well as the inclusion of samples collected across diverse settings. Additionally, to our knowledge, this study represents the first evaluating home testing for GIPs in urine in the pediatric population. Some of these settings and subcategories have small sample sizes. While this may be viewed as a limitation, we aimed to examine GIPs in multiple real-world settings. As such, we found some settings more difficult for subjects to collect samples which is useful for future research. Furthermore, our results would benefit from future expansion of sample collection to include multiple samples for all participants, which could aid in better understanding the relative sensitivity and specificity across different settings/sample collection conditions. Further research to design protocols for general sampling recommendations, given the inter-individual variation in the dynamics of gluten absorption and excretion, will be important to increase the accuracy and utility of GIP detection as a clinical tool.

In summary, we found a small number of children had urine and stool samples with detectable GIPs despite reportedly following a GFD for at least 6 months as a treatment for CeD. The samples were collected across multiple settings, including an endoscopy unit, outpatient gastroenterology clinic, and at home. Additionally, of individuals found to have persistent enteropathy on endoscopy, none had detectable GIPs in their urine, which may relate to the timing of sample collection; timing of gluten exposure, sample collection, and mucosal recovery (ie, insufficient recovery between intermittent gluten exposures), or to nonceliac enteropathy. Further research is needed to better characterize the dynamics of gluten absorption and excretion in pediatric patients, which will help clarify the interpretation of positive or negative GIP results across all collection settings.
